# Surveillance of Noncommunicable Diseases: Opportunities in the Era of Big Data

**DOI:** 10.34133/2022/9893703

**Published:** 2022-06-01

**Authors:** Pengfei Li, Lin Ma, Jue Liu, Luxia Zhang

**Affiliations:** ^1^Advanced Institute of Information Technology, Peking University, Hangzhou 311215, China; ^2^National Institute of Health Data Science, Peking University, Beijing 100191, China; ^3^Peking University Health Science Center, Beijing 100191, China; ^4^Department of Epidemiology and Biostatistics, School of Public Health, Peking University, Beijing 100191, China

## 1. Introduction

Noncommunicable diseases (NCDs) are the leading cause of mortality, accounting for 70% of deaths worldwide, and have become one of the major challenges for human sustainable development in the 21st century [1]. The pandemic of coronavirus disease 2019 (COVID-19) interrupted NCD services in 75% of the countries—public health campaigns and NCD surveillance efforts gave way to pandemic control [2], which further imposes challenges to fight NCDs. Among the key components of strategy for the prevention and control of NCDs, surveillance is a crucial one to track and monitor the major risk factors and intervention effects, which is defined as “the ongoing systematic collection, analysis, and interpretation of health data essential to the planning, implementation, and evaluation of public health practice, closely integrated with timely dissemination of these data to those who need to know” [3, 4]. The capacity to undertake surveillance varied substantially and was inadequate in many low-income and middle-income countries. It is hoped that integration of multiple existing data sources into a comprehensive NCD surveillance framework could enhance the NCD surveillance efforts [5].

## 2. The Current NCD Surveillance System

To guide the implementation of medical and public health measures to control the diseases, Alwan et al. proposed the national surveillance framework for NCDs that encompassed key risk factors, outcomes, health-system response interventions, and health-system capacity [6]. Considering the need of monitoring incidence and the emphasis on representativeness, the periodic population-based survey is one of the most important components of the surveillance system.

The National Health and Nutrition Examination Survey (NHANES) in the United States initiated in the 1960s is one of the early endeavors to monitor the health and nutritional status of nation-wide population. Starting in 1999, NHANES became a continuous program focusing on a variety of health and nutrition measurements each year [7]. Similar population-based surveys have been launched in low-income and middle-income countries (LMICs) with a high burden of NCDs [6]. For example, in China, the nutrition and chronic disease surveys have been combined since 2002 and the resulting China National Nutrition and Health Surveys provide information of “how the social and economic transformation of Chinese society is affecting the health and nutritional status of its population” [8].

Population-based surveys could be extremely manpower, time, and material resource-consuming, limiting NCD surveillance capacity, especially in LMLCs. For example, among 22 LMLCs with a high burden of NCDs, only 50% of them have reported population-based information on risk factors for NCDs [6]. Even for countries with risk factor information, concerns exist regarding the accuracy, quality, and standardization of data [6]. Furthermore, the composition of global burden of diseases has displayed a dynamic trend [9], and the current surveillance system might not have the resilience to include emerging NCDs in a timely manner. For instance, chronic kidney disease (CKD) has only been recognized as an important public health issue worldwide in the past 20 years [10], while the surveillance programs (especially national programs initiated by the government) for CKD are extremely limited [11]. In addition, during major events such as the COVID-19 outbreak [2], risk behaviors affecting health could change rapidly. As a result, the current periodic survey-based system might not be adequate to identify those acute changes. The resource consumption, incomplete reporting, and time lags of existing surveillance systems might damage the ability of governments and health organizations to capture NCD trends and manage unmet needs.

## 3. Big Data and NCD Surveillance Systems

The digitalization in medicine and the advent of big data analytics have introduced novel opportunities for NCD surveillance. The widespread use of electronic health records (EHR), the accumulating administrative and claim data at national level, and other types of datasets have enabled us to pursue solutions to population health issues previously thought impossible [5, 12]. Instead of extrapolating from the data obtained from samples at high costs to make inferences about a population of national level, it is possible to use EHR data across health organizations at the national population level to provide a real-world picture [13]. The practice-driven and longitudinal nature of real-world data enable the inclusion of multiple risk factors, outcomes, and intervention effect evaluation, which are all important elements of NCD surveillance. Furthermore, integrating and analyzing enriched data resources have introduced the feasibility of quick identification and response to changing situations associated with NCD surveillance [14]. As an illustration of the value of multiple data resources, the surveillance of CKD as an emergent NCD is used as an example in the following.

After the concept of CKD was defined in 2002 and existing data revealed its public health importance, the Centers for Disease Control and Prevention in the United States launched the CKD initiative to establish the first comprehensive CKD Surveillance System in 2006 [15]. The approach is to leverage existing data from a wide range of data sources, including data from both healthcare systems and data from existing surveillance programs [11]. Through the integration of various data sources available, the CKD Surveillance System can provide detailed information in support of CKD control in many important aspects.

The similar approach could also be utilized in developing countries with limited resources and capacity. In China, the prevalence of CKD is reported to be 10.8% [16] and imposes a substantial burden on the healthcare system, while CKD was not included in the existing government-initiated surveillance system. Under the national strategy to promote the application of big data, an initiative entitled China Kidney Disease Network (CK-NET) was launched in 2014. CK-NET integrates and analyzes different data source-administrative data, regional EHR, research data, and real-world data, being recognized as the most comprehensive CKD surveillance system and “an important benchmark for kidney disease surveillance in China” [17].

## 4. The Future of NCD Surveillance

Besides big data and data analytics, many cutting-edge technologies could be used to collect data complementing existing NCD surveillance data. As home-based wireless devices, apps, and wearable technology mature in recent years, the real-time health data generated by patients can be collected, which could capture health behaviors including patient engagement [18]. During the pandemic of COVID-19, the cutting-edge technologies are pivotal in infection surveillance. Besides wearable health monitoring sensors, virtual care technologies and Internet-of-Things could be integrated to develop smart disease surveillance systems to prevent, diagnose, and treat COVID-19 [19]. Such kinds of systems could also be instrumental for NCD surveillance [19].

Furthermore, it is increasingly recognized that health is ineluctably linked to social, environmental, and economic factors; hence, including behavioral, environmental, network, and community data into the surveillance of NCDs could lead to opportunities for interventions aimed at improving population health [20]. For example, accumulating evidence suggests that worsening economic outcomes may be a primary contributor to negative health trends among working-age US adults with low income and less education [21]. In addition, virtual digital trails such as mining social media data provide the opportunity to evaluate self-reported NCD-related attitudes and behaviors [5]. Research has shown conveying anger and fatigue on Twitter was associated with an increased risk of heart disease [22]. With the increased complexity of collected data in the surveillance system, tools from other sectors could also be utilized to develop innovative approaches for NCD surveillance. For example, using spatial analyses and geographical information systems provided a geographic perspective to explore health disparities and access to care in patients with head and neck cancer [23].

In summary, the outburst of data in both the medical area and beyond, the development of data analytics and other cutting-edge technologies, and the emergence of data-driven paradigms are transforming NCD surveillance. Although extremely promising, there are still enormous legal, ethical, political, and technical challenges to overcome. In addition, transdisciplinary efforts are unprecedentedly important in the era of big data.
